# AI-Augmented Fundus Disease Screening by Non-Ophthalmologist Physicians: A Paired Before–After Study

**DOI:** 10.3390/bioengineering12121304

**Published:** 2025-11-27

**Authors:** EunAh Kim, Su Jeong Song

**Affiliations:** 1 Department of Ophthalmology, Samsung Changwon Hospital, Sungkyunkwan University School of Medicine, Changwon 51353, Republic of Korea; retinauna@gmail.com; 2 Department of Ophthalmology, Kangbuk Samsung Hospital, Sungkyunkwan University School of Medicine, Seoul 03181, Republic of Korea; 3 Biomedical Institute for Convergence (BICS), Sungkyunkwan University, Suwon 16419, Republic of Korea

**Keywords:** artificial intelligence, decision support, fundus photography, retinal disease screening, primary care, non-ophthalmologist, augmentation, referral triage

## Abstract

Screening for retinal disease is increasingly performed by general practitioners and other non-ophthalmologist clinicians in primary care, especially where access to ophthalmology is limited and diagnostic accuracy may be suboptimal. To investigate the role of an automated fundus-interpretation support solution in improving general physicians’ screening accuracy and referral decisions, we conducted a paired before–after study evaluating an AI-based decision support tool. Four non-ophthalmologists who have been involved in screen fundus images in clinical practice reviewed 500 de-identified color fundus photographs twice—first unaided and, after a washout period, with AI assistance. With AI support, diagnostic accuracy improved significantly from 82.8% to 91.1% (*p* < 0.0001), with the greatest benefit observed in glaucoma-suspect and multi-pathology cases. Clinicians retained final diagnostic authority, and a favorable safety profile was observed. These results demonstrate that AI-assisted diagnosis aid can meaningfully augment non-ophthalmologist screening and referral decision-making in real-world primary care, while underscoring the need for broader validation and implementation studies.

## 1. Introduction

Screening for retinal disease is increasingly performed by general practitioners and other non-ophthalmologist clinicians in primary care [1,2,3,4,5,6,7,8], especially where access to ophthalmology is limited and diagnostic accuracy may be suboptimal. Recent advances in deep learning have led to near-specialist performance in fundus image analysis for a range of retinal diseases, including DR, glaucoma suspect (GS), epiretinal membrane (ERM), retinal vein occlusion (RVO), and macular degeneration (MD) [9,10,11,12,13,14]. For example, Lee et al. developed a deep learning-based decision support tool using 43,221 fundus photographs, achieving a macro-average AUC of 0.964 for five major diseases in external validation, and confirming the utility of independent one-vs-rest classifiers for the detection of multiple co-existing pathologies in real-world screening datasets [14].

Although deep learning systems now demonstrate near-specialist accuracy for fundus image analysis [9,11,12], it remains unclear whether these tools can measurably improve real-world decision-making by non-ophthalmologist physicians, especially under the constraints of routine practice and in cases involving multiple or overlapping pathologies. These studies have primarily examined AI-assisted or autonomous fundus screening performed by non-ophthalmologist clinicians within routine diabetes care, focusing exclusively on DR [9,11,12,15]. We further emphasize that clinical decision-making in ophthalmic care cannot be reduced to a binary DR present/absent judgment [16]. Sight-threatening ocular diseases, including glaucoma, retinal vein occlusion, or age-related macular degeneration, can be present even in eyes without DR [17]. Therefore, non-ophthalmologist clinicians—and any AI tools supporting them—must also be able to recognize and triage such non-DR findings. This broader capability remains underexplored, and our study aims to provide meaningful real-world evidence addressing this important gap.

This study therefore aimed to evaluate whether an artificial intelligence (AI)-based decision support system (Brightics RA, Version 1.0.0; Crystarvision, Seoul, Republic of Korea) could enhance the diagnostic accuracy of non-ophthalmologist clinicians in primary care. The clinical effectiveness of this software was assessed in a real-world workflow, with a particular focus on its impact in complex and multi-pathology cases. Findings from this study may inform best practices for integrating AI decision support into routine retinal screening, supporting a shift toward collaborative “AI plus physician” models of care. Supplementary Table S1 summarizes prior real-world studies evaluating AI-enabled fundus screening.

## 2. Materials and Methods

This paired before–after study was conducted to evaluate the clinical effectiveness of Brightics RA (Version 1.0.0; Crystarvision, Seoul, Republic of Korea), an automated fundus reading software, as a decision support tool for non-ophthalmologist physicians. The overall study design and clinical oversight were coordinated by the Department of Ophthalmology at Kangbuk Samsung Hospital (Seoul, Republic of Korea). The clinical phase of the study was conducted from August 2024 to June 2025, with data analysis performed in July–August 2025. The study protocol was approved by the institutional review board (IRB No.: KBSMC09024). The study was conducted in accordance with the Declaration of Helsinki.

A total of 500 color fundus photographs, categorized by retinal disease status, were obtained from health screening examinees at Kangbuk Samsung Hospital. The image set comprised normal eyes as well as five major retinal diseases: MD, DR, RVO, ERM, and GS. Cases with multiple co-existing pathologies were also included to reflect clinical complexity. All images were anonymized and underwent quality checks to ensure adequacy for analysis and accuracy of labeling. Unreadability was defined as a priori using the Fleming et al. Field Definition Grading Scheme; only Excellent/Good fields were considered gradable [18]. Accordingly, the 99 images graded Inadequate were excluded from the physician alone baseline analysis. However, because suboptimal image quality is common in routine clinical practice, we deliberately underwent subgroup analysis, to observe whether the system could surface clinically actionable signals from images that human readers would otherwise discard. To address potential bias, we added a sensitivity analysis that excludes the same 99 images from both phases. Four non-ophthalmologists (two endocrinologists, one rheumatologist, and one surgeon) participated as study readers. Prior to image interpretation, all readers received a standardized explanation of the disease definitions and labeling criteria for all target retinal diseases. Each clinician independently reviewed the full set of images under two conditions. In the first phase (Phase 1), cases were interpreted in a randomized order without AI support, and diagnostic impressions and confidence scores were recorded. A subset of images assessed as unreadable by the readers was excluded from accuracy calculations, ensuring that only interpretable images contributed to the primary performance metrics.

After a six-week washout period to minimize recall bias, the same set of 500 images was re-evaluated in a new randomized order with the assistance of Brightics RA (Version 1.0.0; Crystarvision, Seoul, Republic of Korea). During this AI-assisted phase (Phase 2), the software provided suggested diagnostic labels, class probabilities, and attention maps for each case. Disease definitions and image labeling were based on the diagnostic criteria and labeling protocol implemented in the Brightics RA (Version 1.0.0; Crystarvision, Seoul, Republic of Korea) AI system [14]. MD was defined according to the criteria of the International Age-Related Maculopathy Epidemiological Study Group, based on the presence of soft drusen (≥63 μm), pigmentary abnormalities, or signs of exudative change [19]. DR was diagnosed if microaneurysms, retinal hemorrhages, hard exudates, or neovascularization were present, following the International Clinical Diabetic Retinopathy Severity Scale [4]. ERM was identified by the presence of a cellophane macular reflex or preretinal macular fibrosis on the fundus image. RVO was determined based on characteristic fundoscopic findings such as venous dilatation, tortuosity, intraretinal hemorrhages, and/or collateral vessel formation. GS was defined by optic disc findings, including a vertical or horizontal cup-to-disc ratio of 0.7 or greater, rim notching or thinning, disc hemorrhage, or a defect in the retinal nerve fiber layer, consistent with International Society of Geographical and Epidemiological Ophthalmology guidelines [20]. Cases with two or more positive findings were categorized as composite (multi-pathology) cases.

**Figure 1:**
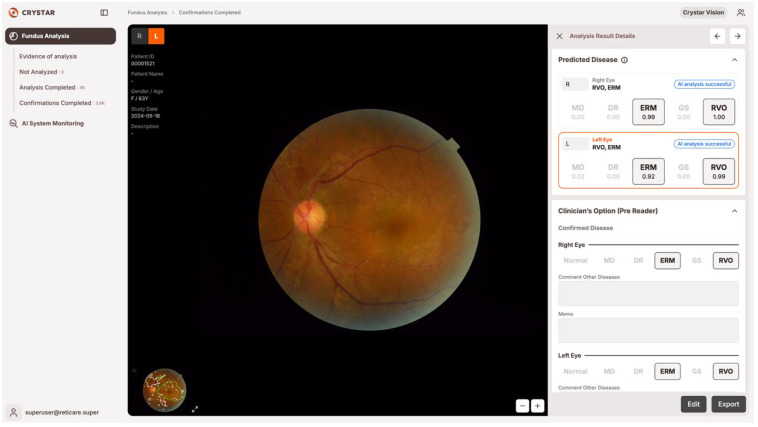
Example of the AI-assisted fundus-interpretation user interface (Brightics RA, Version 1.0.0; Crystarvision, Seoul, Republic of Korea), accessed on 18 May 2024). The platform displays the fundus image with overlaid attention maps for suspected lesions, along with modular one-versus-rest (OVR) classifiers for each disease. The user can review these outputs and make the final diagnostic conclusion.

The Brightics RA (Version 1.0.0; Crystarvision, Seoul, Republic of Korea) system employs modular one-versus-rest (OVR) classifiers for each major retinal disease, as described in our previous work [14]. This OVR architecture enables parallel detection of multiple disease entities in a single fundus image, and facilitates robust performance in the presence of overlapping or complex pathology [14]. For each case, the software highlighted suspected lesion locations with color overlays and presented the predicted probability (range: 0.00–1.00) for each disease category. Physicians could review these visual and numerical cues before making the final diagnostic decision, and were free to ignore or override AI outputs, and final diagnostic decisions remained at their sole discretion in all cases (Figure 1).

The reference standards (“ground truth”) were established by two independent retina specialists (EK and SJS), who were blinded to the study reads. Inter-observer agreement between the two retina specialists is provided in Supplementary Table S2. The AI system was developed and technically supported by Crystarvision AI Research Center. All data were handled in accordance with applicable data protection and medical device regulations.

The primary endpoint was defined as the change in case-level diagnostic accuracy for each reader, with and without AI support, relative to the ophthalmologist reference. Secondary endpoints included disease-specific diagnostic performance, measured by the area under the receiver operating characteristic curve (AUC), and accuracy in cases with multiple co-existing pathologies. Statistical comparisons of paired categorical data were performed using McNemar’s test (Python version 3.10, statsmodels.stats.contingency_tables.mcnemar function), with a two-sided significance level of α = 0.05. Effect sizes were calculated using Cohen’s d, and the number needed to treat (NNT) was estimated from the absolute gain in accuracy for each reader. Net improvement counts were summarized, and exploratory analyses assessed whether benefits concentrated in particular diseases or composite patterns relevant to clinical referral decisions.

To comprehensively assess the impact of AI assistance on physician diagnostic accuracy, we employed multiple complementary metrics that account for the characteristics of our dataset. We quantified false positives (FP) and false negatives (FN) for each pathology category to understand specific types of diagnostic errors, where FP represents cases incorrectly identified as pathological and FN indicates missed diagnoses. For the physician only and physician with AI assistance conditions, four independent readers evaluated all cases, and metrics were reported as mean ± standard deviation to capture both average performance and inter-reader variability.

The F1-score was calculated as the harmonic mean of precision and recall (F1 = 2 × Precision × Recall/(Precision + Recall)), providing a balanced measure that simultaneously considers both types of errors. This metric is particularly suitable for evaluating diagnostic performance where both over-diagnosis and under-diagnosis carry clinical consequences. For the scoring-rule implementation: for single-pathology: correct if the true disease is included, irrespective of additional labels. For multi-pathology: correct only if all ground-truth diseases are included.

Given the inherent class imbalance in our dataset with varying prevalence of different retinal pathologies, we employed Matthews Correlation Coefficient (MCC) as a robust complementary metric [21]. MCC incorporates all four confusion matrix elements (true positives, true negatives, false positives, and false negatives) and produces a balanced measure even when class sizes differ substantially. Unlike accuracy or F1-score, MCC ranges from −1 (complete disagreement) to +1 (perfect prediction), with 0 indicating random performance. This metric has been shown to be more informative than accuracy for imbalanced binary classification tasks, making it particularly valuable for evaluating diagnostic performance across pathologies with different prevalence rates. All metrics were calculated separately for AI only, physician only, and physician with AI assistance conditions.

We adhered to DECIDE AI (Reporting guideline for the early-stage clinical evaluation of decision support systems driven by artificial intelligence) for clinical evaluation of AI-enabled decision support [22]. A completed DECIDE AI checklist is provided in Supplementary Table S3 to facilitate verification of reporting completeness. Because the present work evaluates a fixed, previously validated model (Brightics RA, Version 1.0.0; Crystarvision, Seoul, South Korea) as a clinical aid rather than developing a new prediction model, most TRIPOD AI items related to model development are not applicable; nevertheless, we cross-checked applicable items (model identification/versioning, predictor, and outcome definitions) for consistency within the manuscript.

## 3. Results

The implementation of AI-assisted decision support significantly enhanced the diagnostic accuracy of non-ophthalmologist clinicians in retinal disease screening (Table 1).

Overall diagnostic accuracy improved significantly with AI support across all readers. Case B increased from 42.30% to 63.17% (+20.85%p), Case S from 32.31% to 53.28% (+20.98%p), Case C from 52.52% to 73.40% (+20.86%p), and Case Y from 48.38% to 68.97% (+20.59%p). Mean accuracy across all readers increased from 43.88% to 64.71% (+20.82%p). All improvements were highly statistically significant (*p* < 0.0001). Effect sizes were medium for Case B (Cohen’s d = 0.384) and Case S (Cohen’s d = 0.391), and medium–large for Case C (Cohen’s d = 0.767) and Case Y (Cohen’s d = 0.681). The number needed to treat (NNT) ranged from 5 to 10, indicating that one additional correct diagnosis was achieved for every 5 to 10 cases interpreted with AI support. Case B showed 113 improved cases versus 38 worsened cases, Case S showed 198 versus 83, Case C showed 237 versus 36, and Case Y showed 203 versus 32.

Disease-specific diagnostic performance showed substantial improvement when clinicians used AI assistance (Table 2). Performance was evaluated using F1-score and Matthews Correlation Coefficient (MCC), which provides a balanced measure accounting for all confusion matrix elements and is particularly robust for imbalanced datasets.

For DR, F1-score increased from 0.670 ± 0.043 (physician only) to 0.830 ± 0.150 (physician + AI), with corresponding MCC improvement from 0.578 ± 0.051 to 0.785 ± 0.190. ERM showed marked improvement with F1-score rising from 0.594 ± 0.238 to 0.805 ± 0.156 and MCC from 0.558 ± 0.178 to 0.762 ± 0.179. For GS, one of the most challenging categories for clinicians, F1-score improved dramatically from 0.502 ± 0.257 to 0.804 ± 0.085, with MCC increasing from 0.396 ± 0.305 to 0.745 ± 0.115. MD showed considerable gains with F1-score rising from 0.457 ± 0.200 to 0.696 ± 0.155 and MCC from 0.310 ± 0.255 to 0.606 ± 0.212. RVO demonstrated strong improvement with F1-score increasing from 0.557 ± 0.173 to 0.808 ± 0.167 and MCC from 0.515 ± 0.171 to 0.769 ± 0.196. In contrast, for Normal class, physician + AI achieved F1-score of 0.465 ± 0.164 and MCC of 0.435 ± 0.156, both lower than AI alone (F1-score: 0.151, MCC: 0.160), though this reflects differences in false positive and false negative trade-offs.

Analysis of composite and multi-pathology cases revealed substantial improvements in diagnostic accuracy (Table 3). Case B showed notable gains in composite pathologies: GS + RVO improved from 5.5% to 73.3% (+67.7%p), and ERM + RVO from 0.0% to 65.2% (+65.2%p). Among single pathologies, GS increased from 83.9% to 90.5% (+6.5%p) and ERM from 86.9% to 94.0% (+7.1%p). Case S demonstrated the largest improvements in challenging categories: ERM + GS improved from 3.5% to 45.3% (+41.8%p), and GS + RVO from 4.1% to 32.3% (+28.2%p). For single pathologies, GS showed the greatest gain from 66.7% to 83.1% (+16.5%p), while Normal cases improved from 74.7% to 88.1% (+13.4%p).

Case C exhibited strong performance across pathologies. DR + GS improved from 16.4% to 67.3% (+50.9%p), and GS + RVO from 23.9% to 77.3% (+53.3%p). Single pathologies also showed substantial gains: MD increased from 75.8% to 89.4% (+13.6%p) and ERM from 81.3% to 98.0% (+16.6%p). Case Y showed particularly strong improvements in composite cases. ERM + GS improved from 11.4% to 49.3% (+37.9%p), and GS + RVO from 30.6% to 65.7% (+35.1%p). Among single pathologies, DR demonstrated the largest gain from 76.7% to 96.9% (+20.2%p), followed by MD from 79.7% to 87.5% (+7.8%p).

Across all cases, ERM + GS + RVO consistently achieved 100.0% accuracy with AI support, demonstrating exceptional performance in detecting complex multi-pathology scenarios.

These results indicate that the benefit of AI-assisted decision support was not only consistent across all major retinal disease categories but was especially pronounced in complex cases involving multiple co-existing pathologies and in glaucoma suspect, which are typically challenging for non-specialist clinicians. The marked improvement in these categories underscores the clinical utility of AI in augmenting physician performance in real-world primary care screening settings.

AI support enabled the interpretation of 99 cases that were marked as unreadable by clinicians. These cases were excluded from the primary accuracy analysis in accordance with the final performance report. To ensure that this improvement was not solely driven by these cases, we performed a sensitivity analysis excluding the same 99 images, which yielded consistent results (see Supplementary Table S4).

## 4. Discussion

As highlighted in recent reviews on artificial intelligence in healthcare, evaluating an AI system for clinical use requires careful selection of performance metrics that are tailored to the intended application [23,24,25,26]. Appropriate assessment of AI systems for clinical use should account for diagnostic precision, the ability to identify meaningful pathological features, stability under different imaging conditions, and the practical feasibility of providing timely results within clinical settings [22,23,24,25]. Accordingly, the choice of evaluation methods and endpoints must align with the specific clinical context and use case for which the AI system is intended [23,24,25,26].

Prior evaluations have largely examined AI-assisted or autonomous fundus screening performed by non-ophthalmologist clinicians within routine diabetes care, focusing narrowly on DR [15]. Yet ophthalmic decision-making is not reducible to a binary “DR present/absent” judgment: sight-threatening disease (e.g., glaucoma, retinal vein occlusion, and age-related macular degeneration) can be present even when DR is absent [16]. Real world deployment therefore requires that non ophthalmologist users—and the AI tools supporting them—also recognize and triage non-DR fundus findings [17]. This capability remains underexplored; our study addresses this gap by evaluating AI use by nonspecialist clinicians in routine care and by examining diagnostic performance, image gradeability, and downstream referral outcomes beyond DR alone.

This paired before–after study demonstrated that AI decision support can meaningfully augment the diagnostic accuracy of non-ophthalmologist clinicians in real-world retinal disease screening. Integrating our algorithm (Brightics RA, Version 1.0.0; Crystarvision, Seoul, Republic of Korea) into primary care fundus evaluation workflows yielded a statistically significant improvement in case-level accuracy for all readers (+20.82 percentage points on average), with the most pronounced gains in complex, multi-pathology cases. These results suggest that clinician-in-the-loop AI can strengthen frontline screening performance under routine constraints.

Our findings are consistent with, and extend, prior work on deep learning-based retinal disease screening tools. We previously reported high accuracy for automated detection of DR, ERM, RVO, MD, and GS in a large Korean health screening cohort using modularized OVR classifiers [14]. The present study builds on that foundation by evaluating the same system not as an autonomous reader but as real-time decision support for non-ophthalmologist physicians in a primary care setting, using a paired before–after design. While several studies have examined physicians using AI, most have focused narrowly on AI-assisted or autonomous DR screening within routine diabetes care [15]; in contrast, we assessed multi-pathology decision support in non-specialist workflows, addressing an important evidence gap.

AI assistance improved discrimination across all major retinal disease categories, particularly for DR, GS, RVO, ERM, and MD. The absolute accuracy gains for GS and composite presentations were especially notable, supporting the hypothesis that AI-driven decision support is most valuable in challenging, multi-lesion scenarios where non-specialists face higher cognitive load and greater diagnostic uncertainty. These results imply that deployment of AI tools in frontline can reduce missed referable cases, enhance the appropriateness of referrals to ophthalmology, and shorten time-to-definitive care, thereby avoiding workflow delays and unnecessary retakes [27,28].

Nevertheless, we do need to address several limitations that are observed in this study. First, spectrum (case mix) effects—more variable disease prevalence and severity relative to specialist clinics—tend to depress positive predictive value and increase false positives even when sensitivity is preserved. Second, image acquisition constraints (nonmydriatic pupils, media opacities, operator variability, and limited field) reduce gradeability and can obscure subtle lesions. Third, domain shift between training and deployment (camera model, field of view, illumination, and workflow) can yield uneven sensitivity/specificity for pathologies that were underrepresented during training. Finally, human–AI interaction in non-specialist workflows (automation bias, time pressure, and local referral heuristics) influences how outputs are acted upon and may amplify variability across settings.

The slightly lower AUC for the normal class in the physician + AI condition relative to AI alone likely reflects conservative anchoring to clinical priors—a safety-oriented strategy that minimizes false negatives. While this produced a modest increase in false positive referrals, the trade-off is clinically acceptable when it reduces the risk of missed serious pathology [29]. Notably, the largest gains in complex, multi-pathology cases underscore the complementary strengths of human judgment and AI pattern recognition in realistic diagnostic scenarios.

Our results indicate that AI assistance can help non-ophthalmologist clinicians identify and assess critical structures not only in DR, but also in cases of GS and complex or overlapping pathologies. In particular, the Brightics RA (Version 1.0.0; Crystarvision, Seoul, Republic of Korea) system visually highlights lesion locations on the fundus image and presents quantified probabilities for each disease category, thereby enabling physicians to make informed decisions with greater confidence. By supporting accurate rule-out diagnoses and targeted referral, this approach may enable more efficient allocation of patients to appropriate ophthalmic subspecialists.

There are also several model limitations that we address. First, rare or atypical entities (e.g., myopic maculopathy, retinal dystrophies, and non-glaucomatous disc abnormalities) are under-represented, which can depress sensitivity in the long tail and in multi-pathology presentations. Second, OVR architecture and label interactions. Independent OVR heads can yield incoherent multi-label combinations (e.g., high probabilities for both “normal” and “abnormal” classes or overlapping DR vs. RVO signals) because interclass exclusivity is not explicitly modeled. Per class operating points are calibrated separately, so cross-class trade-offs may not be globally optimal in composite disease cases. Third, while we monitored false negative events for referable disease in general, we acknowledge that subset-specific false negative rates (e.g., multi-pathology strata) were not pre-specified, and we addressed as a limitation.

Additional limitations should be acknowledged. The test dataset originated from a single center with single ethnicity, limiting generalizability. The case mix was fixed and may not capture the full diversity of real-world primary care populations. Outcomes such as decision time, cost-effectiveness, and downstream patient outcomes were not measured. Broader studies are needed to evaluate these aspects and assess fairness and performance across different specialties, imaging devices, and patient populations. Future research should also compare the effectiveness of AI-assisted screening with alternative integration strategies, such as autonomous triage, to further clarify optimal implementation. A planned prospective study will embed the AI into routine care and capture system level metrics (per case decision time, acquisition/retake rates, referral and appointment completion, and time to treatment were not assessed and require prospective implementation studies) and patient relevant outcomes.

## 5. Conclusions

In conclusion, this study provides robust evidence that Brightics RA (Version 1.0.0; Crystarvision, Seoul, Republic of Korea) may meaningfully augment physician expertise in frontline screening for ophthalmic diseases. By delivering real-time support in daily primary care practice, the AI system demonstrated the potential to assist non-ophthalmologist physicians not only in the fundamental task of detecting the presence or absence of disease, but also in identifying specific pathologies and recognizing complex or overlapping conditions. This capability enhances both the accuracy of initial screening and the appropriateness of subspecialty referrals. Primary care clinics can deploy point of care, non-mydriatic fundus imaging in primary care centers, so patients receive an immediate eye exam during the visit; the AI returns a structured normal/referable result within minutes and, when indicated, triggers consult/referral without additional appointments. For urgent care centers and ER: Place portable cameras in urgent centers to triage various eye symptoms and acute vision changes on the spot; AI-assisted readouts guide onsite management versus expedited ophthalmology referral before discharge, minimizing delays and unnecessary returns.

Importantly, our findings reaffirm the conclusions of previous research [30,31], demonstrating that AI is not a replacement for physicians, but rather serves as a valuable adjunct that elevates the accuracy and quality of clinical decision-making. The greatest improvements were observed in diagnostically challenging cases, where AI support enabled more consistent, safer, and more effective referral decisions, while maintaining the physician’s central role in patient management.

To ensure sustainable and generalizable benefits, future implementations should in-corporate comprehensive user training, ongoing threshold calibration, and systematic performance monitoring, including regular audits of false negative rates. Continuous quality assurance, user-driven interface refinement, and multicenter validation will also be essential to maximize clinical impact across diverse real-world environments.

Collectively, these results support a transition from the traditional “AI versus physician” paradigm to a collaborative “AI plus physician” model, empowering physicians to deliver safer, more precise, and more equitable ophthalmologic care within primary care settings.

## Figures and Tables

**Table 1:** Changes in diagnostic accuracy for non-ophthalmologist readers before and after AI assistance. Net improvement counts, number needed to treat (NNT), and effect size are shown. Statistical significance assessed with McNemar’s test.

Reader	Accuracy of AI (%)	Accuracy Without AI (%)	Accuracy with AI (%)	Absolute Gain (%)	Improved Cases	Worsened Cases	NNT	*p*-Value	Effect Size (Cohen’s d)
Case B	84.5	42.30	63.17	+20.85	113	38	5	<0.0001	0.384
Case S	84.5	32.31	53.28	+20.98	198	83	9	<0.0001	0.391
Case C	84.5	52.52	73.40	+20.86	237	36	8	<0.0001	0.767
Case Y	84.5	48.38	68.97	+20.59	203	32	10	<0.0001	0.681
Mean	84.5	43.88	64.71	+20.82					

Note. AI = artificial intelligence; NNT = number needed to treat. “Accuracy without AI” and “Accuracy with AI” refer to overall reader accuracy (%) before and after AI support, respectively. “Absolute Gain” indicates the percentage-point increase in accuracy with AI. “Improved Cases” = number of cases correctly diagnosed only after AI support; “Worsened Cases” = number of cases correctly diagnosed without AI but mis-diagnosed with AI support. *p*-value refers to the statistical significance of the difference in accuracy; Effect Size (Cohen’s d) indicates standardized magnitude of improvement (≈0.2 = small, ≈0.5 = medium, >0.8 = large).

**Table 2:** Performance comparison including false positives (FP), false negatives (FN), F1-score, and Matthews Correlation Coefficient (MCC) for three diagnostic approaches: AI, Physician, and Physician + AI.

Pathology	AI Only			Physician Only			Physician + AI		
	FP/FN	F1-Score	MCC	FP/FN	F1-Score	MCC	FP/FN	F1-Score	MCC
DR	6/6	0.943	0.928	59.5 ± 29.0/22.2 ± 10.4	0.670 ± 0.043	0.578 ± 0.051	21.8 ± 14.5/14.8 ± 17.6	0.830 ± 0.150	0.785 ± 0.190
ERM	3/29	0.837	0.809	29.0 ± 31.3/43.2 ± 35.0	0.594 ± 0.238	0.558 ± 0.178	12.8 ± 5.7/26.0 ± 21.5	0.805 ± 0.156	0.762 ± 0.179
GS	3/34	0.816	0.786	35.0 ± 21.4/58.2 ± 30.7	0.502 ± 0.257	0.396 ± 0.305	30.2 ± 29.0/19.2 ± 4.5	0.804 ± 0.085	0.745 ± 0.115
MD	3/47	0.706	0.684	58.2 ± 25.6/55.0 ± 25.1	0.457 ± 0.200	0.310 ± 0.255	43.8 ± 40.2/29.2 ± 9.6	0.696 ± 0.155	0.606 ± 0.212
Normal	6/39	0.151	0.160	56.0 ± 22.3/18.2 ± 7.5	0.390 ± 0.123	0.333 ± 0.148	18.2 ± 11.1/23.2 ± 9.9	0.465 ± 0.164	0.435 ± 0.156
RVO	15/5	0.896	0.873	15.2 ± 10.7/47.8 ± 19.5	0.557 ± 0.173	0.515 ± 0.171	14.0 ± 9.4/19.0 ± 17.4	0.808 ± 0.167	0.769 ± 0.196

Note. FP = false positives; FN = false negatives; DR = diabetic retinopathy; ERM = epiretinal membrane; GS = glaucoma suspect; MD = macular degeneration; RVO = retinal vein occlusion. Values for “Physician only” and “Physician + AI” represent mean ± standard deviation across all readers. F1-score is the harmonic mean of precision and recall; MCC is a balanced measure of binary classification quality (range: −1 to +1).

**Table 3:** Diagnostic accuracy before and after AI assistance for composite pathology cases.

Pathology	Case B			Case S			Case C			Case Y		
	Accuracy Without AI (%)	Accuracy with AI (%)	Absolute Gain (%p)	Accuracy Without AI (%)	Accuracy with AI (%)	Absolute Gain (%p)	Accuracy Without AI (%)	Accuracy with AI (%)	Absolute Gain (%p)	Accuracy Without AI (%)	Accuracy with AI (%)	Absolute Gain (%p)
DR	89.9	94.8	+4.9	79.5	84.6	+5.1	83.6	96.2	+12.6	76.7	96.9	+20.2
ERM	86.9	94.0	+7.1	80.5	87.8	+7.3	81.3	98.0	+16.6	82.1	97.5	+15.4
GS	83.9	90.5	+6.5	66.7	83.1	+16.5	83.1	91.5	+8.5	89.5	94.1	+4.6
MD	82.8	91.1	+8.3	68.7	70.7	+2.0	75.8	89.4	+13.6	79.7	87.5	+7.8
Normal	91.9	93.2	+1.2	74.7	88.1	+13.4	86.2	91.1	+4.9	88.9	96.7	+7.9
RVO	88.8	98.7	+9.9	84.5	89.4	+4.8	91.1	96.1	+5.0	89.2	93.1	+3.9
DR + ERM	1.2	8.2	+7.0	0.0	28.0	+28.0	51.5	41.5	−10.0	60.6	62.4	+1.8
DR + GS	35.6	44.3	+8.7	3.9	20.1	+16.2	16.4	67.3	+50.9	46.8	65.7	+18.8
ERM + GS	44.1	74.6	+30.5	3.5	45.3	+41.8	52.2	67.7	+15.5	11.4	49.3	+37.9
ERM + RVO	0.0	65.2	+65.2	0.0	13.3	+13.3	48.8	78.5	+29.7	21.3	67.7	+46.4
GS + RVO	5.5	73.3	+67.7	4.1	32.3	+28.2	23.9	77.3	+53.3	30.6	65.7	+35.1
MD + DR	0.0	0.0	0.0	3.6	7.2	+3.6	49.6	52.1	+2.5	44.4	42.9	−1.5
MD + ERM	7.0	27.0	+20.0	0.0	1.9	+1.9	18.0	32.4	+14.3	11.3	24.8	+13.5
MD + GS	0.0	24.3	+24.3	0.0	41.0	+41.0	12.5	35.2	+22.7	4.6	29.4	+24.8
MD + RVO	3.9	0.0	−3.9	0.0	32.4	+32.4	35.3	37.3	+2.0	1.9	5.8	+3.9
ERM + GS + RVO	7.7	99.9	+92.2	0.0	96.0	+96.0	0.0	100	+100	6.7	96.1	+89.4
MD + DR + RVO	89.9	94.8	+4.9	79.5	84.6	+5.1	83.6	96.2	+12.6	76.7	96.9	+20.2

Note. DR = diabetic retinopathy; ERM = epiretinal membrane; GS = glaucoma suspect; MD = macular degeneration; RVO = retinal vein occlusion. Accuracy without AI and Accuracy with AI are expressed in % for each clinician. Absolute Gain (in percentage points, %p) is the increase in accuracy when AI support is used. Mixed pathology rows (e.g., “DR + ERM”) refer to co-existing disease categories.

## Data Availability

Data are available from the corresponding author upon reasonable request.

## Supplementary Materials

The following supporting information can be downloaded at: https://www.mdpi.com/article/10.3390/bioengineering12121304/s1, Table S1: Comparative table with previous studies in real-world evaluations; Table S2: Inter-observer agreement and statistical significance by disease type; Table S3: DECIDE AI checklist; Table S4: Diagnostic accuracy excluding previously unreadable cases (sensitivity analysis).

## Abbreviations

The following abbreviations are used in this manuscript:

DR	Diabetic retinopathy
AI	Artificial intelligence
MD	Macular degeneration
RVO	Retinal vein occlusion
ERM	Epiretinal membrane
GS	Glaucoma suspect
AUC	Area under the receiver operating characteristic curve
NNT	Number needed to treat
