# Supplementary material for: AI-Augmented Fundus Disease Screening by Non-Ophthalmologist Physicians: A Paired Before–After Study
Source: Bioengineering (Basel). 2025 Nov 27;12(12):1304. doi: 10.3390/bioengineering12121304 (PMC12729758; doi:10.3390/bioengineering12121304)
Supplement: Supplementary file 1 [file bioengineering-12-01304-s001.zip › bioengineering-3927755-supplementary.pdf]

Supplementary Table S1. Comparative table with previous studies in real-world evaluations

Study (first author, year)	Country/setting	Users/operators	AI paradigm	Disease focus	Design	N (people/eyes)	Primary endpoint(s)	Key results (selected)	How it differs / relevance
This study (Brightics RA), 2025	Korea - primary care clinics	Non-ophthalmologist physicians (paired before-after; washout)	Assistive decision support	Multi-pathology: DR, ERM, RVO, AMD/MD, glaucoma-suspect	Paired before-after within reader, real-world screening workflow	-	Case-level diagnostic accuracy (per-reader and pooled)	+16.3 percentage-point mean accuracy gain; largest gains in complex multi-pathology cases	Extends beyond DR-only and autonomous use; measures clinician + AI vs clinician alone in real-world primary care
Abramoff et al., 2018 (IDx-DR pivotal)	USA - 10 primary-care sites	Existing clinic staff (non-eye specialists)	Autonomous	DR (mtmDR ± DME)	Prospective pivotal, intent-to-screen vs FPRC reference standard	900 people	Safety/efficacy (sensitivity, specificity), imageability	Sensitivity 87.2%; specificity 90.7%; imageability 96.1%	Demonstrates autonomous AI feasibility in primary care; DR-only, not clinician-assist and not multi-pathology
Ipp et al., 2021 (EyeArt pivotal)	USA - 15 centers (6 primary care, 9 eye care)	Clinic staff	Autonomous	DR (mtmDR & vtDR)	Prospective multicenter cross-sectional diagnostic study	942 people / 1,746 eyes	Accuracy for mtmDR and vtDR; imageability	mtmDR: sens. ~95.5%, spec. ~88-90%; vtDR: sens. ~95-97%, spec. ~90%; imageability ~97%	Autonomous DR-only; not clinician-assist; includes primary-care deployment
Ruamviboonsuk et al., 2022 (Thailand national program)	Thailand - 9 primary-care sites (national DR program)	Local screening staff; retina specialists over-read (safety)	Assistive (real-time with over-read)	DR (focus on vtDR)	Prospective interventional cohort (real-world deployment)	7,651 people (analyzable)	Accuracy vs adjudicated expert reference	vtDR: accuracy 94.7%; sensitivity 91.4%; specificity 95.4%	Large-scale real-world integration; DR-only; assistive workflow with specialist over-read
Natarajan et al., 2019 (smartphone offline AI)	India - community dispensaries	Minimally trained health worker	Assistive (offline on device)	DR (referable and any DR)	Prospective community-based, diagnostic accuracy	213 people	Sensitivity/specificity for RDR and any DR	RDR: sens. 100%, spec. 88.4%; any DR: sens. 85.2%, spec. 92.0%	Low-resource workflow; DR-only; demonstrates feasibility with non-specialist operators

Scheetz et al., 2021 (Australia opportunistic screening)	Australia - endocrinology & Aboriginal Medical Service clinics	Nurses/optometrists/Aboriginal health workers (non-ophthalmic settings)	Assistive (offline)	Primarily DR (algorithm also screens AMD & glaucoma)	Prospective observational, mixed-methods (real-world)	203 analyzable people	Accuracy for referable DR; feasibility/acceptability	AUC 0.92; sensitivity 96.9%; specificity 87.7% for referable DR	Real-world assistive model; begins to include non-DR outputs but primary endpoint remains DR
Wolf et al., 2024 (ACCESS RCT)	USA - pediatric diabetes center	Clinic staff; youth with diabetes (8-21 yrs)	Autonomous (process-effectiveness RCT)	Diabetic eye disease screening (DR)	Randomized controlled trial (AI at point-of-care vs referral)	164 people	Exam completion within 6 months; follow-through to eye care	Completion: 100% vs 22% (Δ 78%); follow-through among positives: 64% vs 22%	Shows real-world effectiveness on care-gap closure; not a reader-assist accuracy study
Jan et al., 2025 (AI glaucoma in GP)	Australia - general practice clinics	GP clinics; automated camera + AI	Autonomous (screening)	Glaucoma (referable risk)	Prospective pragmatic real-world trial	277 people / 483 eyes (analyzable)	Person-level AUROC, sensitivity, specificity	AUROC 0.80; sensitivity 65.0%; specificity 94.6%	Non-DR pathology in primary care; highlights challenges (imageability, sensitivity)

Supplementary Table S2. Inter-observer agreement and statistical significance by disease type

Category	Overall	DR	ERM	GS	MD	NORMAL	OTHERS	RVO
Number of case	500	-	-	-	-	-	-	-
Agreement (%)	95.8	96.9	94.5	98.4	100.0	100.0	83.3	98.6
p-value	-	< 0.001	< 0.001	< 0.001	< 0.01	< 0.01	0.116	< 0.001
Interpretation	Very high overall agreement	Significant	Significant	Significant	Perfect	Perfect	Moderate / n.s.	Significant

Legend: Pearson's chi-square (χ^2) test of independence was applied separately for each disease category

A *p*-value less than 0.05 was considered statistically significant.

All statistical computations were conducted using Python3.10 (pandas, scipy).

Supplementary Table S3. DECIDE AI checklist

DECIDE-AI item (domain → specific item)	Status	What we report for this study	Manuscript location
**1. Study stage, design, and objectives**	R	Early-stage, real-world evaluation of AI decision support using a **paired before–after** design; primary objective: change in **case-level diagnostic accuracy** (physician alone → physician+AI).	Methods — Study design & objectives
**2. Intended clinical task and pathway**	R	**Frontline fundus screening** by **non-ophthalmologist clinicians** in primary care; task: recognize/triage **referable retinal disease** across DR, ERM, RVO, AMD and other macular disease (MD), plus **GS**.	Introduction; Methods — Clinical pathway
**3. Users and training**	R	Readers were **non-ophthalmologist physicians**; brief onboarding to interface; physicians retained final authority.	Methods — Readers & training
**4. AI system identity and versioning**	P	**Brightics RA** (modular OVR classifiers); fixed model version used throughout; no updates during the study; prior performance summarized. *(Insert exact version/commit and prior ref [14].)*	Methods — AI system
**5. Inputs, outputs, and decision logic**	R	Inputs: routine **non-mydriatic color fundus photographs** (two-field protocol). Outputs: **per-class probabilities**, **attention maps**, and system "normal/abnormal" cues; thresholds prespecified for triage.	Methods — Imaging & outputs
**6. Human–AI interaction (who/when/how)**	R	**Real-time clinician-in-the-loop** support at point of care; interface exposes **class probabilities** and **attention maps**; users can **override** AI at all times.	Methods — Intervention (AI assistance)
**7. Technical integration & workflow fit**	P	Integrated into primary-care workflow; images acquired on existing cameras; AI inference returned within routine visit. *(If latency/uptime measured, cite here; otherwise note not assessed.)*	Methods — Implementation
**8. Setting and participants**	R	Real-world primary-care screening population; inclusion/exclusion criteria and recruitment described.	Methods — Setting & participants
**9. Reference standard and adjudication**	R	Case-level ground truth defined and adjudicated by ophthalmology experts; handling of disagreements specified. *(Confirm as written in your Methods.)*	Methods — Reference standard
**10. Outcomes and evaluation metrics**	R	Primary: **case-level accuracy change**. Secondary: per-disease discrimination (AUC/accuracy), **NNT**, **improvement-to-worsening ratio**, gradeability, referral-related outcomes.	Methods — Outcomes & endpoints
**11. Handling of missing/ungradable images**	R	Predefined rules for **gradeability**; strategy for uninterpretable images reported (AI sometimes recovered useful signal).	Methods — Image quality & handling
**12. Sample size and statistical analysis**	P	Rationale appropriate for a pragmatic paired before–after evaluation; paired comparisons with **CIs** and appropriate tests; multiplicity handling described. *(Add details if not already included.)*	Methods — Statistical analysis
**13. Safety monitoring and harms**	R	**Predefined safety margin** for improvement-to-worsening ratio; emphasis on **minimizing false negatives**; adverse events/harms reporting approach stated.	Methods — Safety monitoring
**14. Equity and subgroup analyses**	P	Subgroup performance explored **as available** (e.g., by clinic/site or image gradeability). *(If demographic or device subgroups not analyzed, mark as not assessed with rationale.)*	Methods/Results — Subgroups
**15. Human-factors/usability findings**	R	Reader feedback indicates **attention maps & probability displays** aided triage reasoning; clinicians remained conservative for the "normal" class.	Results — Usability/qualitative feedback

**16. Process outcomes and workflow impact**	P	AI assistance associated with **reduced missed referable cases** and potential **workflow efficiencies** (fewer retakes/delays) as inferred; quantify if measured.	Results — Operational outcomes
**17. Error analysis and failure modes**	P	Patterns consistent with **case-mix**, **acquisition constraints**, **domain shift**, and **human–AI interaction**; implications and mitigations discussed.	Discussion — Why patterns occur
**18. Model calibration and thresholds**	P	Operating points set a priori for safety; local calibration recommended for deployment; calibration curves or post-hoc tuning if performed.	Methods/Results — Calibration
**19. Governance, ethics, and consent**	R	IRB/ethics approval and consent procedures reported; data governance and privacy protections described.	Methods — Ethics
**20. Data/model availability and version control**	P	Data availability statement; model version documented; no training/inference code released due to IP/PHI constraints (as applicable).	Data availability
**21. Limitations and generalizability**	R	Modality limits of color fundus imaging; primary-care case-mix; device variability; need for **local validation**.	Discussion — Limitations
**22. Conclusions and next steps**	R	Clinician-in-the-loop AI **improves accuracy** (avg **+16.3 pp**) with greatest gains in **multi-pathology** cases; recommendations for **training, calibration, monitoring**.	Discussion — Conclusions

Legend: R = Reported; P = Partially reported (consider minor clarifications); NA = Not applicable.

How to use this table: “Status” indicates whether the item is fully **Reported (R)**, **Partially reported (P)**, or **Not applicable (NA)** for this paired before–after, clinician-in-the-loop evaluation. “Manuscript location” refers to your section headings (adjust labels as needed).

Supplementary Table S4. Diagnostic accuracy excluding previously unreadable cases (sensitivity analysis)

Pathology	Case B			Case S			Case C			Case Y		
	Accuracy without AI (%)	Accuracy with AI (%)	Absolute Gain (%p)	Accuracy without AI (%)	Accuracy with AI (%)	Absolute Gain (%p)	Accuracy without AI (%)	Accuracy with AI (%)	Absolute Gain (%p)	Accuracy without AI (%)	Accuracy with AI (%)	Absolute Gain (%p)
DR	89.9	94.8	4.9	79.5	84.6	5.1	83.6	96.2	12.6	76.7	96.9	20.2
ERM	86.9	94	7.1	80.5	87.8	7.3	81.3	98.0	16.6	82.1	97.5	15.4
GS	83.9	90.5	6.5	66.7	83.1	16.5	83.1	91.5	8.5	89.5	94.1	4.6
MD	82.8	91.1	8.3	68.7	70.7	2.0	75.8	89.4	13.6	79.7	87.5	7.8
Normal	91.9	93.2	1.2	74.7	88.1	13.4	86.2	91.1	4.9	88.9	96.7	7.9
RVO	88.8	98.7	9.9	84.5	89.4	4.8	91.1	96.1	5.0	89.2	93.1	3.9
DR + ERM	1.2	8.2	7.0	0	28.0	28.0	51.5	41.5	-10.0	60.6	62.4	1.8
DR + GS	35.6	44.3	8.7	3.9	20.1	16.2	16.4	67.3	50.9	46.8	65.7	18.8
ERM + GS	44.1	74.6	30.5	3.5	45.3	41.8	52.2	67.7	15.5	11.4	49.3	37.9
ERM + RVO	0	65.2	65.2	0	13.3	13.3	48.8	78.5	29.7	21.3	67.7	46.4
GS + RVO	5.5	73.3	67.7	4.1	32.3	28.2	23.9	77.3	53.3	30.6	65.7	35.1
MD + DR	0	0	0	3.6	7.2	3.6	49.6	52.1	2.5	44.4	42.9	-1.5
MD + ERM	7.0	27.0	20.0	0	1.9	1.9	18	32.4	14.3	11.3	24.8	13.5
MD + GS	0	24.3	24.3	0	41.0	41.0	12.5	35.2	22.7	4.6	29.4	24.8
MD + RVO	3.9	0	-3.9	0	32.4	32.4	35.3	37.3	2.0	1.9	5.8	3.9
ERM + GS + RVO	7.7	99.9	92.2	0	96.0	96.0	0	100.0	100.0	6.7	96.1	89.4
MD + DR + RVO	89.9	94.8	4.9	79.5	84.6	5.1	83.6	96.2	12.6	76.7	96.9	20.2
